# Strategies to support implementation of an intervention embedding healthy eating and activity content within community-based home visiting

**DOI:** 10.3389/frhs.2026.1951390

**Published:** 2026-10-14

**Authors:** Rachel G. Tabak, Amanda Gilbert, Alexandra B. Morshed, Sarah S. Farabi, Cynthia D. Schwarz, Allison Kemner, Debra Haire-Joshu

**Affiliations:** 1 Prevention Research Center, Washington University in St. Louis Bursky School of Public Health, St. Louis, MO, United States; 2 Center for Diabetes Translation Research, Washington University in St. Louis Bursky School of Public Health, St. Louis, MO, United States; 3 Department of Behavioral, Social, and Health Education Sciences, Rollins School of Public Health, Emory University, Atlanta, GA, United States; 4 Goldfarb School of Nursing at Barnes-Jewish College, St. Louis, MO, United States; 5 Center for Human Nutrition, Washington University in St. Louis School of Medicine, St. Louis, MO, United States; 6 Parents as Teachers National Center, St. Louis, MO, United States

**Keywords:** dissemination, implementation and sustainability, healthy eating, home-visiting, implementation science, physical activity

## Abstract

**Introduction:**

Embedding evidence-based health interventions within community-based home visiting models offers opportunities to leverage the expertise of these organizations to support reach, trust, and sustainability. This is particularly the case for mothers facing barriers to healthcare access. This study used a Designing for Dissemination and Sustainability approach and the Implementation Strategies Applied in Communities (ISAC) compilation to describe implementation strategies and perspectives of these strategies to support a healthy eating and active living intervention (HEALTH) embedded within Parents as Teachers (PAT), a national community-based home visiting model serving families from pregnancy through early childhood.

**Methods:**

Implementation determinants were informed by prior mixed-methods work and stakeholder engagement with the PAT National Center, affiliate staff (supervisors and parent educators), and annual conference participants. An anonymous 5-item survey administered at the 2025 PAT Annual Conference assessed stakeholder perceptions of proposed implementation strategies. Participants rated strategies on perceived impact and feasibility and reported role and community type. Descriptive analyses were conducted.

**Results:**

Forty-nine participants from 18 states completed the survey; most were parent educators. Across strategies, training on implementing the intervention and materials to support supervisors for reflexive supervision were rated as the most impactful and feasible. Communities of practice, parent educator team meetings, and resource connection supports were viewed as highly impactful but somewhat less feasible. Ratings were generally similar across rural and mixed or urban settings, and parent educators often rating strategies as more feasible than supervisors or leaders.

**Discussion:**

Findings suggest that leveraging existing organizational infrastructure, especially training, reflective supervision, and peer learning supports, may facilitate implementation of health-related interventions in community settings. Stakeholder input was essential for tailoring strategies to workforce role and context. These results can inform future implementation planning for HEALTH and similar interventions embedded in community-based organizations.

## 1 Introduction

Embedding intervention content related to health into the existing programs and services community-based organizations deliver builds on the infrastructure of these organizations to reach those who may benefit most and on their deep expertise in supporting clients and families (1). Further, it leverages the expertise of these organizations in meeting people where they are; these organizations are able to develop rapport and trust (relational connections) and sustained contact. This also addresses the barriers to accessing healthcare, particularly for communities bearing the greatest chronic disease burdens (2–7). Additionally, healthcare systems and providers structured around traditional medical care and billing face significant challenges adapting to building trusted relationships (8, 9). Yet many community-based organizations are not designed to deliver health-related interventions (10). To support the impact of health-related interventions in practice, it is therefore important to use an intentional approach to developing interventions and implementation strategies (11–20).

Implementation science is a science that aims to understand the deliberate spread and uptake of interventions and programs into wider use in usual care settings. As such, it is well positioned to support embedding health-related intervention content within community-based organizations (21). Furthermore, implementation science describes implementation strategies (22–24), the methods designed to enhance the adoption, implementation, and scale-up of evidence-based interventions. The implementation strategies applied in communities (ISAC) compilation identifies strategies, specifically for community-based organizations (22). Further, implementation science provides frameworks, measures, and methods to guide assessment of contextual determinants (e.g., barriers and facilitators) at multiple levels (e.g., individual, provider, organization), termed ‘implementation context’. These tools can improve understanding of factors that influence how an intervention is delivered in the real world, can help embed health-related interventions with community-based organizations, and can help develop and tailor implementation strategies matched with implementation context (25).

A 2025 review highlighted opportunities to apply implementation science and a Designing for Dissemination and Sustainability (D4DS) approach in community settings (26). This is largely because both approaches emphasize engaging community organization partners from the earliest stages of intervention development (26). D4DS aligns health-related interventions and implementation strategies with the usual practice and reimbursement structure of the organization by incorporating the perspectives of partners, including participants, providers, and organizational leadership, and assuring fit within the existing infrastructure (13, 16, 17).

Parents as Teachers (PAT) is a national evidence-based home visiting model implemented through a diverse network of locally administered affiliates. PAT National Center serves as the model developer and national intermediary organization, providing curriculum, training, technical assistance, implementation guidance, quality improvement supports, and professional development. Local affiliates deliver the model within community settings such as schools, nonprofit organizations, early childhood agencies, and public systems. PAT is supported through federal, state, and local funds and is a voluntary program with all services provided free of charge. Of the families PAT serves, 82% have at least one stressor defined by factors such as low educational attainment, low income, recent immigrant family, or unstable housing (27). In 2024-2025, PAT affiliates served more than 77,000 families and 93,000 children nationally (27). The PAT evidence-based model is delivered by a global network of affiliates and parent educators. PAT National Center provides support for affiliates to meet requirements as well as further quality standards that represent best practices in the field. PAT affiliates are located in a variety of settings (e.g., social service non-profits, schools, early childhood organizations). In 2024-2025, PAT had 858 affiliates in the United States and was delivered by 4,300 parent educators (27).

Our team has partnered with PAT to develop, embed, and evaluate a healthy weight intervention (Healthy Eating and Active Living Taught at Home, HEALTH) in its routine practice. The team has further demonstrated the effectiveness of HEALTH to prevent weight gain; the evidence-based intervention (28–31). A 24-month group-randomized, nested cohort trial demonstrated the effectiveness of HEALTH on metrics key to preventing chronic diseases with 230 mothers with overweight or obesity and their preschool children (32–35). By 24 months, mothers receiving HEALTH lost on average −1.5 kg, while mothers receiving usual care gained 3.2 kg (*p* = .002) (28). Further, a 24-month randomized trial tested an adapted HEALTH for pregnancy/postpartum, and found that among 267 pregnant Black mothers enrolled in PAT, 89% of whom participated in Medicaid, mothers receiving HEALTH had reduced one-year postpartum weight retention (2.5 kg vs. 5.7 kg, *p* = .01) (29–31). As is recommended by a Designing for Dissemination approach, HEALTH was developed to be embedded in PAT, to support future implementation, sustainability, and scale up (13, 16, 36). This is well aligned with several ISAC strategies [Table 1, pre-dissemination strategies].

**Table 1:** Implementation strategies employed in designing for dissemination approach to HEALTH development.

ISAC Strategy	Definition
Change adaptable program components	Tailor the program through fidelity-consistent changes to meet the needs of the priority population or setting
Facilitate implementation	Offer assistance on the implementation process through an interactive, supportive relationship
Design pragmatic programs	Develop programs that are low cost, easy to implement, and relevant to multiple populations
Use technology for evaluation	Use or develop apps, websites, or databases to facilitate improve data collection and sharing
Meet community partners’ needs	Select or adapt programs to respond to community partners’ needs, concerns, or resources
Use reimbursement systems	Advocate for or establish systems for payors (insurers, employers) to reimburse EBI costs
Engage community members	Share information about the EBI and seek community member input
Plan for sustainability	Set up funding structures to support program maintenance, and work with implementing sites to provide resources and support to continue the EBI
Build partner relationships	Invest time to build long-term relationships with community partners (e.g., PAT stakeholders)
Develop structured curriculum	Develop structured curriculum, including lessons plans and other content, for program implementers
Develop adaptable programs	Ensure programs are adaptable to meet the needs of organizations and priority populations

HEALTH, Healthy Eating and Active Living Taught at Home; PAT, Parents as Teachers; EBI, Evidence-based intervention

Studies to implement HEALTH within a small subset of PAT sites across the country employed several implementation strategies and allowed for extensive assessment of implementation determinants. This work, conducted before the development of the ISAC, employed implementation strategies suggested by a more clinically oriented compilation of implementation strategies (Expert Recommendations for Implementing Change, ERIC) (22, 23, 37). The strategies included: *develop and distribute educational materials* including HEALTH intervention materials to deliver to families and training materials; *make training dynamic* via interactive training through a synchronous web-based experience; and *provide ongoing consultation* via technical support (38). Qualitative, quantitative, and mixed-methods investigation identified that determinants at multiple levels (e.g., individual, provider, and organization) are important determinants (e.g., barriers and facilitators) of implementation, and require additional implementation strategies (39–42). These eleven determinants include parent educator experience and level of comfort with discussing weight and HEALTH content, supervisor and peer support, perceived value, fit with workflow, relationships and trust, and mother social needs.

The current study uses D4DS and ISAC to describe the implementation strategies identified to support a health-related intervention within a community setting and provider perceptions of the importance and feasibility of these strategies.

## 2 Materials and methods

### 2.1 Community-based implementation setting

The community-based organization for the current study is Parents as Teachers (PAT), which is a maternal-child home visiting organization in the United States (43). PAT is an award-winning national organization that serves families from pregnancy until the youngest child in the home enters kindergarten to build strong communities, thriving families and children who are healthy, safe, and ready to learn. Nearly 40 years of independent research has found that PAT is effective in achieving the model goals (44). Additionally, through decades of partnered research, including with members of the present research and PAT teams, PAT has effectively incorporated healthy eating and activity behaviors to prevent chronic disease among families they serve (45). However, a gap remains in understanding how implementation of this content can be supported.

PAT National Center, located in St. Louis, MO, is responsible for training and certifying parent educators (via extensive web-based training), developing new and updating existing curricula, providing implementation guidance, ongoing professional development, and continuous quality improvement supports to affiliates. In addition to formal training systems, PAT National Center supports implementation through implementation support specialists, reflective supervision resources, technical assistance, communities of practice, webinars, conferences, peer learning opportunities, and ongoing dissemination of evidence-informed practices. These structures provide a national infrastructure for supporting adoption, implementation, and sustainment of new content and innovations across a diverse network of affiliates.

### 2.2 New implementation strategy development

To identify and specify strategies to address the determinants uncovered and support the next stage of translation of this research to practice, the team engaged in a series of meetings with the PAT National Center team. This allowed the research and PAT National Center teams to develop strategies that leveraged ongoing, successful approaches employed by the organization, as suggested by Balis et al. (46). Rather than creating entirely new implementation supports, strategy development intentionally focused on leveraging and strengthening existing PAT implementation infrastructure. Aligned with the approach described in Balis et al. (2023) (47), we conducted a series of meetings between the research team and teams at PAT National Center (e.g., research and learning, implementation support, training, curriculum) iteratively matching determinants that were identified in previous research with potential strategies from ISAC. Through this process, proposed strategies were designed to align with structures already familiar to affiliates, including reflective supervision, implementation support, professional development, peer learning opportunities, communities of practice, webinars, and conference-based learning. This approach was intended to maximize fit with existing organizational workflows while minimizing burden on local programs and parent educators. Additional approaches to solicit perspectives from parent educators and other stakeholders (e.g., funders, supervisors, state leadership) included presentations by the research team and PAT National Center partners at the Annual PAT Conference in 2023, 2024, and 2025. During these workshops, the team presented determinants and engaged in discussions about potential strategies. These conversations, workshops, and meetings yielded an initial set of strategies, which are specified in Table 2.

**Table 2:** Implementation strategies to support HEALTH implementation.

ISAC strategy	Definition	Strategy	Actors	Action	Temporality & dose	Determinant
Facilitate peer learning	Set up networking and collaborating opportunities for implementers to learn from peers	PE implementation team meetings	Supervisor PEs	PEs within an affiliate meet to share successes, challenges implementing HEALTH	After initial training, staff meeting cadence varies by site (e.g., weekly)	PEs uncomfortable delivering HEALTH PEs run out of time to deliver HEALTH
Facilitate peer learning	Set up networking and collaborating opportunities for implementers to learn from peers	Communities of Practice (CoP)	PATNC Supervisor PEs	PEs meet across affiliates, topics determined by CoP	Monthly, after initial training	PEs uncomfortable delivering HEALTH PEs run out of time to deliver HEALTH
Provide resources	Develop and share resources to enhance program delivery	Supervisor materials for reflexive supervision (RS)	Supervisors observe, provide feedback, HEALTH supports	Developed by PATNC Supervisor uses materials during RS meetings with PEs	After initial training; any RS meeting; frequency determined by supervisor	Supervisor capacity PEs feel unsupported in delivering HEALTH PEs uncomfortable delivering HEALTH
Provide training	Train staff, volunteers, or partner organizations on the EBI	Ongoing/ refresher training opportunities	PATNC training team, research team develop trainings	Development of brief (<20 min) online refresher trainings to address common challenges/ solutions	Ongoing Number determined by need, informed by modeling	PEs uncomfortable delivering HEALTH Specific parts require additional training (e.g., discussing weight)
Create program guide	Create a playbook, blueprint, or guide describing how to implement the EBI	HEALTH-focused impl. support calls	PATNC impl. support team Supervisor	Impl. support team member and supervisor problem solve challenges to HEALTH implementation	After initial training, HEALTH focused calls 1x/month (same cadence as typical calls)	Supervisor capacity Affiliate is having difficulty accessing HEALTH materials Staff turnover
Provide technical assistance	Offer guidance (including through external organizations) on implementing an EBI	Increase training for impl. support team	PATNC impl. support team Research team	Problem solve and develop additional resources and training	Ongoing, quarterly check-ins determine if resources are needed	impl. support team unsure how to support supervisors New implementation challenges arise
Provide resources	Develop and share resources to enhance program delivery	Tips developed by PEs, delivered through PATNC social media	PEs PATNC impl. support team	PEs develop short messages/videos with implementation tips PATNC shares via social media	Ongoing, at least quarterly	PEs uncomfortable delivering HEALTH PEs run out of time to deliver the intervention
Engage potential partners	Use networking, relationships, or outreach events to share information about the EBI with potential partners	HEALTH specific podcast specific series	PATNC engagement team	Develop podcast series about HEALTH and how to access training	Monthly	PEs, supervisors unaware of HEALTH PE, supervisors do not see HEALTH as valuable
Facilitate peer learning	Set up networking and collaborating opportunities for implementers to learn from peers	Short messages and videos about HEALTH	PATNC engagement team	PEs develop short messages/videos about HEALTH, PATNC shares with links to access training	Monthly	PEs, supervisors unaware of HEALTH PE, supervisors do not see HEALTH as valuable
Engage potential partners	Use networking, relationships, or outreach events to share information about the EBI with potential partners	Webinars about HEALTH	PATNC engagement team	PATNC presents webinar about HEALTH and how to access training	2–4x/yr	PEs, supervisors unaware of HEALTH PE, supervisors do not see HEALTH as valuable

ISAC, implementation strategies applied in communities; HEALTH, Healthy eating and active living taught at home; PE, parent educator; PAT, parents as teachers; PATNC, PAT national center; EBI, evidence-based intervention; IMPL., implementation, CoP, communities of practice; RS, reflexive supervision.

### 2.3 Potential impact and feasibility of implementation strategies

To determine PAT stakeholder ratings of the potential impact and feasibility of the preliminary implementation strategies, an anonymous survey was administered during the Annual PAT Conference (San Juan, PR) in 2025.

Participants/Respondents: Slides with a Quick Response (QR) code to the survey were displayed during workshops conducted by the research team and PAT National Center team members; this was the only recruitment strategy. There were no inclusion/exclusion criteria; anyone with the QR code could respond to the survey. To indicate consent to participate, participants responded Next to the item: “I have read and understand the above consent form. By clicking the “Next” button to enter the survey, I indicate my willingness to voluntarily take part in this survey.”

Data collection and analysis: A complete version of the survey, which included five items, is included in Supplementary file 1. Respondents were asked to rate the potential impact and feasibility of the strategies (response options: Not at all; Somewhat; Moderately; Considerably; Extremely; Don't know) as well as to report their role in PAT (e.g., parent educator, supervisor), and the type of geographic area (e.g., rural, urban) served by their PAT site. Reponses to potential impact and feasibility were dichotomized such that %Extremely/considerable impact were combined as were Moderately, Somewhat, and not at all (%Moderately/somewhat/not at all); ‘Don't know’ was considered missing. These were calculated for the Total sample and subgroups of participants (i.e., “Parent educators”, “Supervisors/Leaders”). Descriptive statistics and comparisons by PAT role and geographic region of the respondent were conducted in Stata. Statistical testing was not conducted due to the sample size and potential for multiple comparisons, but differences between respondent types were considered meaningful if they were ≥20%. This difference was determined to be substantively meaningful for description to the research and community organization teams.

## 3 Results

### 3.1 Participants

Forty-nine participants responded to the survey. Most participants (*n* = 29, 62%) were parent educators, while the remaining were in supervisory or leadership roles. Respondents were from 18 states; the state with the largest number of participants (*n* = 14, 29%) was Illinois, while several states had only one participant (e.g., Connecticut, Georgia, Michigan). Participants identified a range of communities served. Most (*n* = 37, 77%) served a mix of communities including suburban, urban, and rural or only urban communities, but 23% (*n* = 11) served only rural and/or small town communities.

### 3.2 Rating

There was a broad range of ratings across the different strategies in the survey (Figure 1). The strategy with the highest percentage of respondents (*n* = 41, 89%) rating the potential impact as extremely or considerably likely to have potential impact was ‘training on implementing the intervention’; this strategy also had the second highest percentage of respondents (*n* = 38, 83%) rating the potential feasibility as extremely or considerably feasible. ‘Materials to support supervisors for reflexive supervision’ was the strategy with the highest percentage of respondents rating the feasibility as extremely or considerably feasible, and this strategy also had the second highest percentage of respondents rating the strategy as extremely or considerably impactful. The strategies with the lowest percentage of respondents rating them as extremely or considerably likely to have potential impact and feasibility were ‘podcast series focused on the intervention’ and ‘implementation support calls focused on the intervention’.

**Figure 1:**
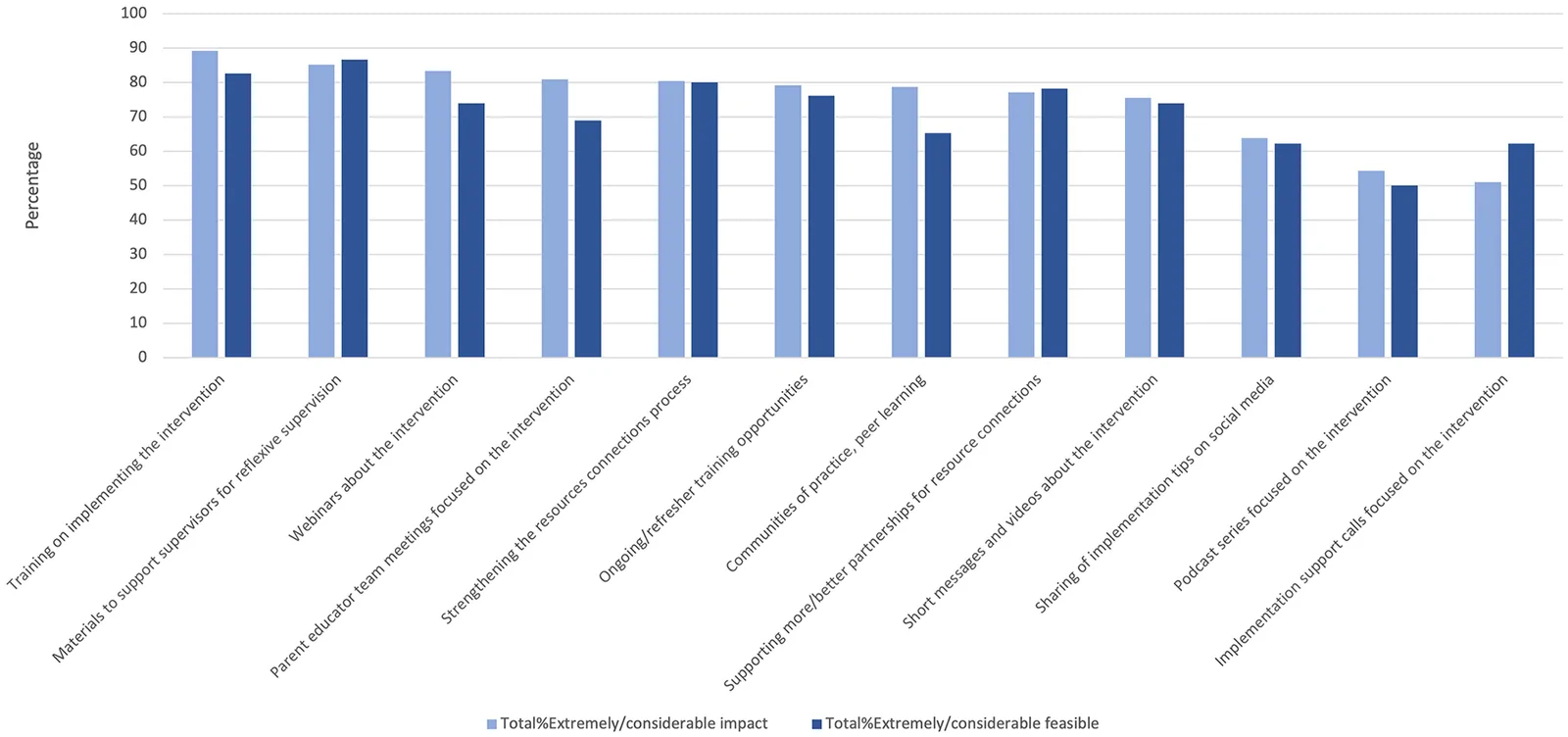
Percent of participants rating each strategy as extremely/considerable impact of feasible.

### 3.3 Ratings by role and community served

The ratings for impact and feasibility were similar (no difference >20% between participants serving larger/mixed and rural/small town communities, data not shown). For several strategies, the percentage of parent educators rating for impact and feasibility differed by more than 20% (Figure 2). While 94% (*n* = 16) of respondents in supervisor/leader roles rated 'Short messages and videos about the intervention' as extremely or considerably impactful, only 65% (*n* = 17) of those in parent educator roles had this rating. However, for ‘podcast series focused on the intervention’ and ‘implementation support calls focused on the intervention’ 67% (*n* = 18) and 59% (*n* = 16) of participants in parent educator roles rated them as extremely or considerably impactful, but this was only the case for 41% (*n* = 7) and 39% (*n* = 7) for participants in supervisor/leader roles. These two strategies were also rated differently between respondents in parent educator roles, with the percentage rating them as extremely or considerably feasible higher (62%, *n* = 15and 72%, *n* = 18) for parent educators than for supervisor/leaders (37%, *n* = 6% and 41%, *n* = 7).

**Figure 2:**
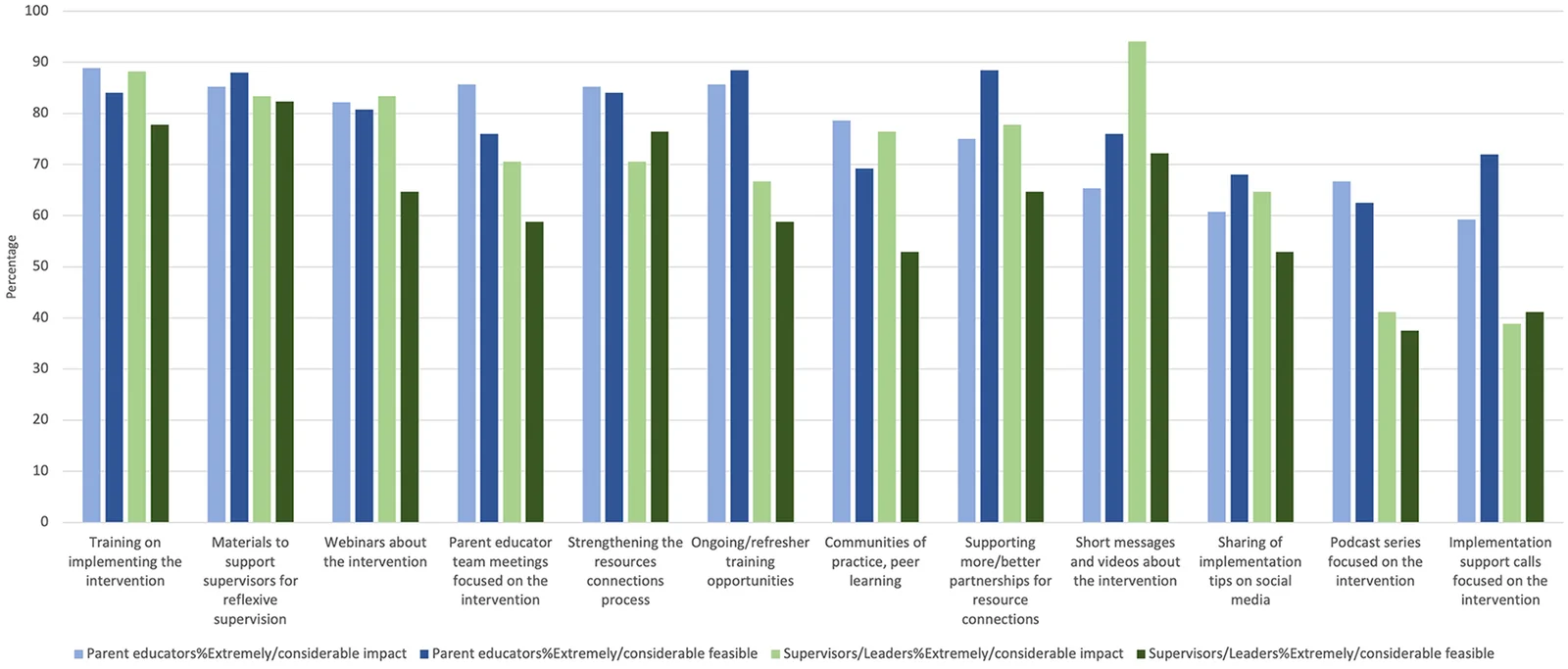
Percent of participants rating each strategy as extremely/considerable impactful or feasible by role.

For several other strategies (‘Ongoing/refresher training opportunities’ and 'Supporting more/better partnerships for resource connections'), the percentage of parent educators who rated them as extremely or considerably feasible was more than 20% higher than the percentage of supervisor/leaders (Figure 2). While 88% (*n* = 23) of participants in parent educator roles rated these strategies as extremely or considerably feasible for both strategies this percentage was only 59% (*n* = 10) and 65% (*n* = 11) among participants in supervisor/leadership roles, respectively.

## 4 Discussion

This paper used a D4DS approach and the ISAC compilation to describe the implementation strategies identified to support a health-related intervention within a community setting. Parent educator and supervisor/leader perceptions suggest ‘training on implementing the intervention’ and ‘Materials to support supervisors for reflexive supervision’ have the greatest potential for impact and feasibility (13, 16, 17, 22). When exploring differences greater than 20%, this analysis found a greater percentage of parent educators rated nearly all strategies as extremely/considerable impactful/feasible than supervisors/leaders. However, this study did not find meaningful differences between respondents who reported working in rural/small towns and those working in all other community types (e.g., urban, suburban). This may be, in part, because the strategies were part of PAT National Center's existing infrastructure rather than creating entirely new strategies; the D4DS process supported alignment with organizational processes.

In community settings, implementation strategies focused at levels beyond individual practitioners are likely to be more effective than those limited to only the provider level (26, 48–50). Findings in the current study suggest there are differences between parent educators and supervisors/leaders in their ratings. These findings point to the selection of strategies that may be uniquely suited to each level (i.e., provider and supervisor/leader). Supporting more/better partnerships for resource connections as ‘extremely or considerably more feasible than supervisors’ did, additional work may be needed to build support among supervisors. Within PAT, these findings suggest that investments in workforce supports, reflective supervision, technical assistance, and peer learning may provide benefits beyond implementation of a single intervention. These existing structures create opportunities to integrate new evidence-based interventions and innovations over time, allowing programs to respond to emerging family and community needs without requiring entirely new implementation supports for each initiative. More broadly, these findings may be relevant for other community-based organizations. Organizations with established implementation supports may be better positioned to integrate multiple evidence-based interventions over time by leveraging existing relationships, workforce development systems, and quality improvement structures rather than creating intervention-specific supports for each new initiative.

Several other studies have investigated key stakeholders' perceptions of implementation strategies (51–54), often as part of refining strategies before testing. Kamran et al. used an engaged, phased approach to map implementation strategies to barriers and facilitators. They then solicited feedback on strategy acceptability and feasibility from multiple perspectives (i.e., patients, partners, healthcare providers) before making iterative modifications to finalize the strategy set (51). Knapp et al. used a survey to allow a Stakeholder Advisory Panel to rate each strategy's perceived effectiveness, feasibility, and priority for their community health center. Like the current study, they also found training and leadership support to be key strategies for supporting implementation, in their case of a pediatric hypertension clinical practice guideline implementation. Unlike the current study, they also identified the importance of strategies embedded in medical-records (e.g., reminders, order sets), which may be more relevant to the clinical context than the community setting in which the current study was conducted (53).

Other studies have also explored whether perceptions of implementation strategies differ based on implementation context. Among primary care physicians Glasgow et al. found little difference between the providers based on rural/urban differences or other provider or organizational context (54). Similarly, LeLaurin et al. conducted a study to understand implementation of social needs interventions using the electronic medical record. They used focus groups, interviews, and workflow assessments with providers and staff at pediatric clinics and also found that ratings for acceptability and appropriateness were higher than feasibility (55). In the current study, we also found that for most strategies, feasibility was rated as lower than potential impact.

### 4.1 Limitations

This is a descriptive study, with a sample size that limits statistical testing as there was no pre-specified power analysis and many of the subgroups explored included small numbers. The cross-sectional design would also limit the interpretation of statistical comparisons based on respondent characteristics. Further, while there is considerable diversity in representativeness of the participants to PAT programs across the US, this survey was conducted at the Annual Conference, which may not be accessible to all programs. Thus this convenience sample includes self-section and assessed perceptions of implementation strategies, rather than implementation outcomes. Notwithstanding these limitations, this study adds to a growing literature showing how employing D4DS in developing and improving implementation strategies in our previous work led to implementation strategies that key stakeholders perceived as feasible and potentially impactful, as shown in the current survey.

### 4.2 Implications

This work suggests applying a D4DS approach can help develop an intervention with improved fit to a setting, but that using taxonomies for implementation strategies specific to the implementation context, such as ISAC may offer additional strategies. Further, considering the perspectives of key stakeholders in deciding what strategies to include in implementation efforts is key as there is considerable variability in how strategies are perceived in terms of potential impact and feasibility.

## Data Availability

The datasets used/analyzed during the current study are available from the corresponding author on reasonable request. Requests to access the datasets should be directed to Rachel Tabak, rtabak@wustl.edu.
